# 肺癌肺上叶切除术后单管胸腔闭式引流的回顾性分析

**DOI:** 10.3779/j.issn.1009-3419.2019.03.07

**Published:** 2019-03-20

**Authors:** 强 柏, 春全 刘, 永 崔

**Affiliations:** 100050 北京，北京友谊医院胸外科 Department of Thoracic Surgery, Beijing Friendship Hospital, Beijing 100050, China

**Keywords:** 胸腔闭式引流术, 肺上叶切除术, 肺肿瘤, Closed chest drainage, Lung lobectomy, Lung neoplasms

## Abstract

**背景与目的:**

目前，肺上叶切除术后留置单管胸腔引流还是双管胸腔引流管仍存在争议，本研究对因肺癌行肺上叶切除术、常规淋巴结清扫术后留置单根胸腔闭式引流管患者的术后引流相关并发症进行统计分析，评价引流效果。

**方法:**

回顾性分析2012年4月-2017年5月入住北京友谊医院胸外科因肺癌行肺上叶切除术、常规淋巴结清扫术后放置单根引流管患者的临床资料，评价单根胸腔闭式引流管的引流效果。

**结果:**

301例患者行肺上叶切除术、常规淋巴结清扫术后放置单根胸腔引流管，术后并发症发生率为9.3%，其中胸腔引流管相关并发症占5.64%。

**结论:**

肺上叶切除术、常规淋巴结清扫术后单管胸腔闭式引流的引流效果不亚于双管引流。

## Abstract (en)

**Background and objective:**

There is no consensus on one or two chest drains closed chest drainage in superior lobectomy of lung cancer and mediastinal lymph node dissection. This study investigated the postoperative complications about drainage and evaluated the effectiveness.

**Methods:**

We retrospectively reviewed the clinical data of patients with single closed chest drainage in superior lobectomy of lung cancer and mediastinal lymph node dissection of Beijing Friendship Hospital between April 2012 and May 2017, and evaluated the effectiveness.

**Results:**

From the available data of 301 patients, the complication rate after superior lobectomy was 9.3%, and the complication rate of drainage after superior lobectomy was 5.64%.

**Conclusion:**

The effectiveness of drainage of single closed chest drainage in superior lobectomy of lung cancer and mediastinal lymph node dissection is no less than double drainage.

目前，肺叶切除术后留置单管或是双管胸腔闭式引流仍存在争议，传统上双管居多，逐步采用单管快速康复、避免不必要的损伤和刺激、减少疼痛是胸腔闭式引流的主要目的。我院传统上即广泛采用单管。有学者认为肺叶切除术后留置单管胸腔闭式引流可达到充分引流的效果，并有相关文献报道肺叶切除术后单管胸腔闭式引流的效果不亚于双管引流。本文将结合国内外研究进展和我们工作中的体会，对因肺癌行肺上叶切除术、常规淋巴结清扫术后放置单根胸腔引流管的引流情况及相关并发症进行回顾性分析，评价单管胸腔闭式引流的引流效果。

## 1 资料与方法

### 1.1 研究对象

收集于2012年4月-2017年5月入住北京友谊医院胸外科因肺癌行肺上叶切除术+淋巴结清扫术后放置单根引流管的患者，共341例。筛选后共有301例患者纳入本研究。

### 1.2 入选标准

所有患者在手术前均未接受过放疗、化疗或免疫治疗，近期无肺炎病史，无免疫缺陷病史；排除其中40例资料不全者。

### 1.3 胸腔引流管放置方法

28号胸腔引流管（直径9.3 mm），头端放至胸膜顶，侧孔距离胸壁5 cm处。

### 1.4 资料搜集

搜集患者年龄、性别、术后日均引流量、术后带管时间、术后住院天数、术后引流相关并发症等相关临床资料，并进行整理、列表。

### 1.5 统计学处理

采用SPSS 20.0统计软件进行分析，计量资料采用均数±标准差（Mean±SD）表示，采用*t*检验。计数资料采用率（%）表示，采用卡方检验。以*P* < 0.05为差异有统计学意义。

## 2 结果

### 2.1 患者一般资料

301例患者中，男性206例，女性95例，平均年龄（61.50±9.61）岁。见表 1-表 2。

**1:** 301例患者一般临床资料及引流相关情况 Clinical characteristics and results of 301 patients enrolled in the study

Clinical characteristics	Data
Male	206
Female	95
Age (yr)	61.50±9.61
Mean of amount of drainage everyday (mL)	202.78±122.96
Duration of chest tube drainage (d)	6.87±5.60
Length of stay after pulmonary lobectomy (d)	10.24±5.82

**2:** 301例患者年龄分布 Age distribution of 301 patients enrolled in the study

Age (yr)	*n*
24-29	1
30-39	2
40-49	24
50-59	93
60-69	120
70-79	54
80-85	7

### 2.2 术后并发症

术后发生并发症的患者共28例（9.3%），包括肺不张17例（5.64%），其中因需要放置第二根引流管者2例（0.66%），皮下气肿7例（2.32%），拔管后再置管1例（0.33%），术后血胸1例（0.33%），术后因病情加重死亡1例（0.33%），术后乳糜胸3例（0.99%），术后脓胸2例（0.66%），心律失常5例（1.66%），其中阵发性房颤1例，阵发性室早二联律1例，心前区不适1例（既往冠心病病史，心电图未见明显异常），窦性心动过速2例，术后再次手术者1例（0.33%；因血胸），术后行支气管镜下吸痰3例（0.99%）。见表 3。

**3:** 301例患者出现术后并发症的资料分析 Postoperative complications of 301 patients enrolled in the study

Complications	*n* (%)
Atelectasis	17 (5.64)
Need for additional chest tube	2 (0.66)
Subcutaneous emphysema	7 (2.32)
Drain reinsertion	1 (0.33)
Hemothorax	1 (0.33)
Mortality	1 (0.33)
Chylothorax	3 (0.99)
Asperaive bronchoscopy	3 (0.99)
Pleural empyema	2 (0.66)
Arrthymia	5 (1.66)
Reoperation	1 (0.33)

### 2.3 术后引流相关情况

301例患者术后日均引流量为（202.78±122.96）mL，平均带管时间（6.87±5.60）d，平均术后住院时间（10.24±5.82）d。

## 3 讨论

本文着重从术后平均住院时间、日平均引流量、术后带管时间、术后引流相关并发症的发生率来探讨肺上叶切除术、常规淋巴结清扫术后单管引流的优劣性。

### 3.1 单管引流相对于双管引流并未增加术后平均住院时间

由表 1可知，肺上叶切除术、常规淋巴结清扫术后平均住院天数为（10.24±5.82）d，有研究^[1-11]^比较肺叶切除术后单管引流和双管引流差异的临床试验结果显示，肺上叶切除术后单管引流相对于双管引流并未增加术后平均住院时间。Okur等^[12]^的100例肺叶切除术后单管引流和双管引流的实验结果显示：单管引流与双管引流在术后平均住院时间方面无统计学差异。

### 3.2 单管引流相对于双管引流并未增加日平均引流量，甚至少于双管引流

本研究中，日平均引流量为（202.78±122.96）mL。有研究^[1, 2]^比较肺叶切除术后单管引流相对于双管引流差异的临床试验结果显示：肺叶切除术后单管引流相对于双管引流并未增加日平均引流量。而Okur等^[12]^的100例肺叶切除术后单管引流和双管引流的实验结果显示：单管引流量[（600.0±43.2）mL]明显少于双管引流[（896.0±56.2）mL]（*P* < 0.001）。结合本科室经验，单管引流相对于双管引流对胸膜的刺激更小，胸水分泌减少，可在一定程度上减少胸腔引流量。具体机制及程度有待进一步探讨。甚至有研究^[13-20]^认为术中探查术侧肺部水试验无漏气，可尽量避免留置胸腔引流管，解除引流管对胸腔的刺激，从而减少术后胸水的产生，缩短住院时间。

### 3.3 单管引流相对于双管引流并未增加术后带管时间

本研究中术后带管时间（6.87±5.60）d。有研究^[1, 2]^结果显示：肺上叶切除术后单管引流相对于双管引流并未增加术后带管时间。Okur等^[12]^的实验结果也显示肺上叶切除术后单管引流相对于双管引流并未增加术后带管时间。Abel Go´mez-Caro等^[3]^在肺叶切除术后留置单管和留置双管对患者术后影响的随机对照研究结果显示：肺叶切除术后单管引流相对于双管引流并未增加术后带管时间。

### 3.4 单管引流相对于双管引流并未增加术后引流相关并发症的发生率

本研究中，术后共有17例患者出现引流相关并发症（5.64%）。Abel Go´mez-Caro等^[3]^在肺叶切除术后单管引流和双管引流对患者术后影响的随机对照研究结果显示，肺叶切除术后单管引流相对于双管引流的术后并发症的发生率并未增加（两组术后引流相关并发症均为5%），与本研究的结果无统计学差异（*P*=1.0）；本研究中共有28例（9.3%）患者出现术后并发症，相比Abel Go´mez-Caro等在术后双管引流研究中术后并发症的发生率（18.6%）存在差异（*P*=0.035），单管引流的术后并发症的发生率更低，可以认为术后单管引流不亚于双管引流。有研究^[4-10]^在对肺叶切除术后单-双管引流效果的临床观察*meta*分析中的结论显示，即肺叶切除术后放置单管引流并未增加引流相关并发症的发生率。

关于肺叶切除术后单管引流与双管引流效果及引流相关并发症的文献分析均提示肺上叶切除术后单管引流效果相当或优于双管引流。Icard等^[11]^对100例肺叶切除术后患者进行单管胸腔引流的实验研究表明，无一例患者需要再次手术或置管，90%患者能按时拔管，认为肺叶切除术后单管引流的有效性和安全性不亚于双管引流。

Pawelczy等^[13-15]^对183例肺叶切除术后患者的临床观察研究发现，单管引流与双管引流在引流量、术后支气管镜吸痰和再次置管、术后相关并发症及死亡率方面均无统计学差异；单管引流患者住院时间显著短于双管（7.6 d *vs* 9.0 d; *P*=0.001）。

因国家、地区不同，放置引流管的习惯、方法、拔除胸腔引流管的标准不一，导致术后带管时间、术后住院时间、术后并发症的发生率存在差异。个别患者因术中分离组织粘连导致术后局部渗血，引流增加，从而使患者带管时间、术后住院时间延长，也使术后引流相关并发症的发生率升高，但在相同研究标准下的临床观察显示：肺上叶切除术后单管引流的有效性、安全性和术后引流相关并发症方面不亚于双管引流。

总之，肺癌肺上叶切除术后单管引流效果不亚于双管。本研究的缺陷：单臂回顾性研究。

因术后肺炎的诊断标准不一，病情程度及病灶面积变异较大，本文并未将术后肺炎纳入研究范围。从统计结果不难看出，术后肺不张的发生率远较其他相关并发症高，说明影响术后肺不张发生的因素较多，除充分引流排气外，还需患者有良好的依从性，强调患者教育的重要性，配合临床大夫进行早期的术后康复锻炼。

肺叶切除术后应用胸腔闭式引流已成为一种规范，尽管很重要，但目前发表的相关文献却为数不多，而实际上大多数胸外科中心都在应用单管引流。目前，通过阅读相关文献及结合本科室的临床经验得出的结论有：①单管引流临床应用的优势包括：有利于患者术后活动和物理康复训练、引流管刺激胸膜引起的胸水减少、患者舒适度和依从性增加、利于皮肤切口愈合等，从而降低术后平均住院时间、日平均引流量、术后带管时间、术后引流相关并发症的发生率；②置管时，主张置于胸顶且于低位开侧孔，③若临床把握适当，其引流效果优于或相当于双管引流，且不增加并发症的发生率和死亡率。

## References

[1] Alex J, Ansari J, Bahalkar P (2003). Comparison of the immediate postoperative outcome of using the conventional two drains versus a single drain after lobectomy. Ann Thorac Surg.

[2] Kim SS, Khalpey Z, Daugherty SL (2016). Factors in the selection and management of chest tubes after pulmonary lobectomy: Results of a national survey of thoracic surgeons. Ann Thorac Surg.

[3] Abel Gomez-Caro A, Roca MJ, Torres J (2006). Successful use of a single chest drain postlobectomy instead of two classical drains: a randomized study. Eur J Cardiothorac Surg.

[4] Zhou D, Deng XF, Liu QX (2016). Single chest tube drainage is superior to double chest tube drainage after lobectomy: a *meta*-analysis. J Cardiothorac Surg.

[5] Drahush N, Miller AD, Smith JS (2016). Standardized approach to prolonged air leak reduction after pulmonary resection. Ann Thorac Surg.

[6] Royer AM, Smith JS, Miller A (2015). Safety of outpatientchesttube management of air leaks after pulmonary resection. Am Surg.

[7] Rocco G, Brunelli A, Rocco R (2017). Suction or nonsuction: How to manage a hest ube fter ulmonary esection. Thorac Surg Clin.

[8] Filosso PL, Sandri A, Guerrera F (2017). Management ofchestdrainsafterthoracic resections. Thorac Surg Clin.

[9] Li SJ, Zhou K, Li YJ (2017). Efficacy of the fissureless technique on decreasing the incidence of prolonged air leak afterpulmonary lobectomy: A systematic review and *meta*-analysis. Int J Surg.

[10] Yap KH, Soon JL, Ong BH (2017). The safe volume threshold for chest drain removal following pulmonary resection. Interact Cardiovasc Thorac Surg.

[11] Icard P, Chautard J, Zhang X (2006). A single 24 F blake drain after wedge resection or lobectomy: a study on 100 consecutive cases. Eur J Cardiothorac Surg.

[12] Okur E, Baysungur V, Tezel C (2009). Comparison of the single or double chest tube applications after pulmonary lobectomies. Eur J Cardiothorac Surg.

[13] Pawelczyk K, Marciniak M, Kacprzak G (2007). One or two drains after lobectomy? A comparison of both methods in the immediate postoperative period. Thorac Cardiovasc Surg.

[14] Tanaka M, Sagawa M, Usuda K (2014). Postoperative drainage with one chest tube is appropriate for pulmonary lobectomy: a randomized trial. Tohoku J Exp Med.

[15] Jiang H, Wang J, Yuan DF (2015). Feasibility and safety of early chest tube removal after complete video-assisted thoraciclobectomy. Indian J Cancer.

[16] Koç T, Routledge T, Chambers A (2010). Do patients undergoing lung biopsy need a postoperative chest drain at all?. Interact Cardiovasc Thorac Surg.

[17] Satherley K, Luckraz H, Rammohan KS (2009). Routine placement of an intercostal chest drain during video-assisted thoracoscopic surgical lung biopsy unnecessarily prolongs in-hospital length of stay in selected patients. Eur J Cardiothorac Surg.

[18] Tsukioka T, Takahama M, Nakajima R (2015). Thoracic drainage using nasal airway for postoperative broncho-pleural fistula. Kyobu Geka.

[19] Yang M, Fan J, Zhou HX (2015). What are the advantages? A prospective analysis of 16 versus 28 french chest tube sizes in video-assisted thoracoscopic surgery lobectomy of lung cancer. Zhongguo Fei Ai Za Zhi.

[20] Filosso PL, Sandri A, Guerrera F (2017). Management of chest drains after thoracic resections. Thorac Surg Clin.

